# Combination of Iron and Zinc Enhanced the Root Cell Division, Mitotic Regularity and Nucleolar Activity of Hexaploid Triticale

**DOI:** 10.3390/plants12132517

**Published:** 2023-06-30

**Authors:** Ana Carvalho, Alexandra Lino, Carolina Alves, Catarina Lino, Débora Vareiro, Diogo Lucas, Gabriela Afonso, José Costa, Margarida Esteves, Maria Gaspar, Mário Bezerra, Vladimir Mendes, José Lima-Brito

**Affiliations:** 1 Plant Cytogenomics Laboratory, Department of Genetics and Biotechnology, University of Trás-os-Montes and Alto Douro (UTAD), 5000-801 Vila Real, Portugal; 2 Centre for the Research and Technology of Agro-Environmental and Biological Sciences (CITAB), Institute for Innovation, Capacity Building and Sustainability of Agri-Food Production (Inov4Agro), UTAD, 5000-801 Vila Real, Portugal; 3 University of Trás-os-Montes and Alto Douro, 5000-801 Vila Real, Portugal; lino.alexandra@hotmail.com (A.L.); carolinasantosalves31@gmail.com (C.A.); catarina.lino@outlook.com (C.L.); adelaidemarafona@hotmail.com (D.V.); diogo.lucas_@hotmail.com (D.L.); gabrielaafonso96@gmail.com (G.A.); jliuze@gmail.com (J.C.); anamargaridaesteves12@gmail.com (M.E.); mariatxi.gaspar@gmail.com (M.G.); mariojbezerra02@gmail.com (M.B.); vlamendesp@gmail.com (V.M.)

**Keywords:** chromosomal anomalies, micronutrients, nucleolus, seed priming, triticale

## Abstract

Hexaploid triticale results from crosses between durum wheat and rye. Despite its high agronomic potential, triticale is mainly used for livestock feed. Triticale surpasses their parental species in adaptability and tolerance to abiotic and biotic stresses, being able to grow in acidic soils where a high amount of iron (Fe) and zinc (Zn) is typical. On the other hand, high amounts of these essential trace elements can be cytotoxic to bread wheat. The cytotoxicity induced by seed priming with a high concentration of Fe and Zn impaired root cell division and induced nucleolar changes in bread wheat. Such cytogenetic approaches were expedited and successfully determined cytotoxic and suited micronutrient dosages for wheat nutripriming. With this study, we intended to analyse the hexaploid triticale cv ‘Douro’ root mitotic cell cycle and nucleolar activity after seed priming performed with aqueous solutions of iron (Fe) and/or zinc (Zn), containing a concentration that was previously considered cytotoxic, to bread wheat and to infer the higher tolerance of triticale to these treatments. The overall cytogenetic data allowed us to conclude that the Fe + Zn treatment enhanced the root mitotic index (MI), mitosis regularity and nucleolar activity of ‘Douro’ relative to the control and the individual treatments performed with Fe or Zn alone. The Fe + Zn treatment might suit triticale biofortification through seed priming.

## 1. Introduction

Biofortification can increase the Fe and Zn contents of edible parts of plants, contributing to the eradication of the hidden hunger caused by the deficiency of these essential trace elements in the human diet [1]. Stangoulis and Knez [1] reviewed different biofortification approaches developed for the last 25 years in major crops such as wheat, rice, maize and barley, as well as the encouraging results and benefits to human and animal health. Among the challenges described to achieving stable biofortified crops, the authors reported climate change and the inherent occurrence of biotic and abiotic stresses that can induce variations in the Fe and Zn contents [1]. Additionally, among the biofortification strategies to ensure effective crop biofortification in the future, the authors reported that plant breeders have been crossing Fe- and Zn-dense progenitors with parents of good agronomic performance to develop large populations which, upon selection, can guarantee the maintenance of nutrient-dense genotypes through the generations [1]. Beyond the biofortification through the soil and foliar application reported by [1], seed nutripriming, which consists of the imbibition of seeds in micronutrient-rich solutions before germination, constitutes an additional strategy widely used in cereals [2,3,4]. In bread wheat, biofortification with Fe and/or Zn through seed priming [5] or application on soil and leaves improved the nutritional value of whole wheat flour [6]. Additionally, the benefits of seed priming with Fe and/or Zn were perceivable in the untreated offspring of the biofortified plants [7].

Hexaploid triticale (× *Triticosecale* Wittmack; AABBRR; 2n = 6× = 42) is a synthetic amphiploid that results from crosses between durum wheat (*Triticum turgidum* subsp. *durum*, AABB, 2n = 4× = 28) and rye (*Secale cereale* L., RR, 2n = 2× = 14). Triticale grain is mainly used as livestock feed, given its high biomass and yield [8,9], which usually surpass those of its parents [10]. In general, triticale merges the advantages of its parental species—being highly productive and resilient and having biochemical and technological properties—with the potential to industry and use as a new food source [9,10,11]. Although triticale can grow in acidic and dry soils, any given cultivar can be considered stress-tolerant *a priori* [10]. Triticale tolerates soils with low pH, but this feature is accompanied by unbalanced concentrations of iron (Fe) and zinc (Zn), which are essential to biological processes, plant growth and development, and animal and human diets and health. Triticale has been less exploited at the biofortification level than the major crops. Triticale seeds primed with distilled water (hydropriming), mineral solutions or melatonin showed improvement in germination, root and seedling growth and/or tolerance to drought and salt osmotic stress [3,12,13,14]. Nevertheless, considering its main application as livestock feed, its resilience and its cross-ability with other species of the tribe Triticeae, the biofortification of triticale with Fe and/or Zn could be interesting in the future.

Different triticale varieties were more tolerant to high concentrations of zinc (Zn) and/or iron (Fe^3+^) under hydroponics than wheat and rye [15,16]. Camargo et al. [15] studied the root growth of triticale, wheat and rye exposed to high Fe and/or Zn concentrations but did not analyse cytogenetic effects.

There is a need to define balanced micronutrient dosages to be used in the nutripriming of grain cereals to avoid deficiency or toxicity. Analytical methods and techniques that can provide fast results in plant nutrition towards the definition of the best biofortification strategy or dosage for each crop or variety are highly required. Previously, we analysed how various concentrations of Fe and/or Zn used in bread wheat seed priming impacted root cell division and nucleolar activity [5,17,18]. Roots are growing organs in direct contact with soil or another substrate, and when exposed to excess micronutrients, their cell division is impaired and their growth is compromised due to the generated cytotoxicity [17,19,20]. The nucleolus is an organelle that senses the intracellular stress caused by drought, salinity or excess micronutrients, responding with changes in number, morphology, area and structure [5,19,20,21,22]. Besides, the nucleolar activity directly implies that protein synthesis is highly required for vital biological processes and determines food quality [5,7].

Triticale can tolerate higher Fe and/or Zn concentrations than wheat and rye [15]. Hence, for this work, we hypothesised that using a dosage of Fe and/or Zn that was somewhat cytotoxic to bread wheat when used previously in seed priming [17] would not be cytotoxic to hexaploid triticale. Therefore, with this study, we aimed to analyse the triticale cv. ‘Douro’ (2n = 6× = 42) root mitotic cell cycle and nucleolar activity upon seed priming performed with aqueous solutions containing 8 mg·L^−1^ of Fe and/or Zn and to infer the higher tolerance of triticale to these treatments.

The global cytogenetic results evidenced that the combined use of 8 mg·L^−1^ Fe + 8 mg·L^−1^ Zn for priming of ‘Douro’ se stimulated the root mitotic index (MI), the nucleolar activity and increased the mitosis regularity. Additionally, this seed priming treatment was less cytotoxic to triticale than to bread wheat and might be suitable for triticale’s biofortification.

## 2. Results

### 2.1. Mitotic Cell Cycle Analysis

The number of interphases and dividing cells per observation field, preparation and treatment were variable. In total, we scored 1975 cells (1299 interphase + 676 dividing cells) in control, 1311 cells (584 interphase + 727 dividing cells) in the Fe treatments, 802 cells (630 interphase + 172 dividing cells) in the Zn treatments, and 3939 cells (3212 interphase + 727 dividing cells) in the Fe + Zn treatments.

**Figure 1:**
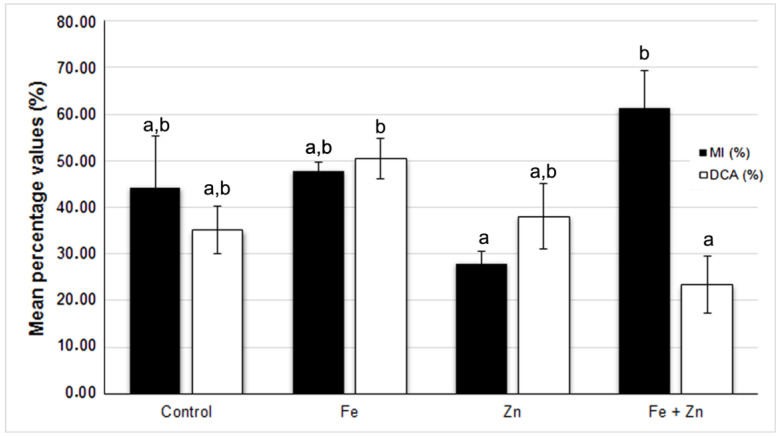
Mean percentage values (±S.E., error bar) of mitotic index (MI) and dividing cells with anomalies (DCA) per seed priming treatment performed in the triticale cv. ‘Douro’ seeds. Different lowercase letters among MI or DCA columns represent statistically significant differences (*p* ˂ 0.05).

The highest mean percentage value of the mitotic index (MI) was detected in the roots of seeds primed with Fe and Zn, which differed significantly (*p* ˂ 0.05) from those treated only with Zn, which showed the lowest mean value of MI (Figure 1).

The highest mean percentage value of dividing cells with anomalies (DCA) was shown by roots from seeds primed only with Fe that differed significantly (*p* ˂ 0.05) from those treated with Fe and Zn, which presented the lowest average value of DCA (Figure 1).

**Figure 2:**
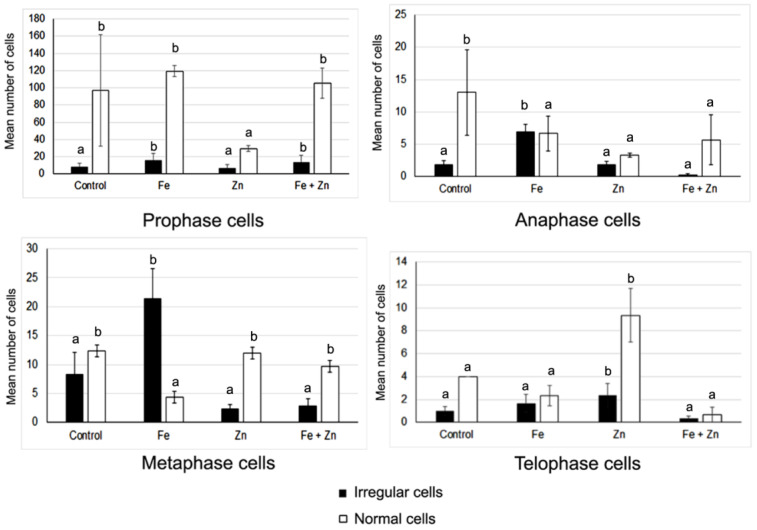
Mean number (±S.E., error bar) of dividing cells into the different mitotic phases determined per seed priming treatment performed in the triticale cv. ‘Douro’ seeds. Different lowercase letters in the columns of the same data series (normal or irregular cells) represent statistically significant differences (*p* ˂ 0.05) among treatments.

Concerning the dividing cells in the different mitotic phases, the mean number of normal cells was higher than the average of irregular ones (Figure 2).

The two exceptions occurred for the mean number of irregular anaphases and metaphases scored in the Fe treatment (Figure 2). Most of the dividing cells were in prophase and metaphase, indicating cell cycle arrest in these mitotic phases (Figure 2). On the other hand, the Zn treatment showed the lowest mean values of normal and irregular prophase cells and the highest average of normal telophase cells (Figure 2), suggesting mitosis completion.

**Figure 3:**
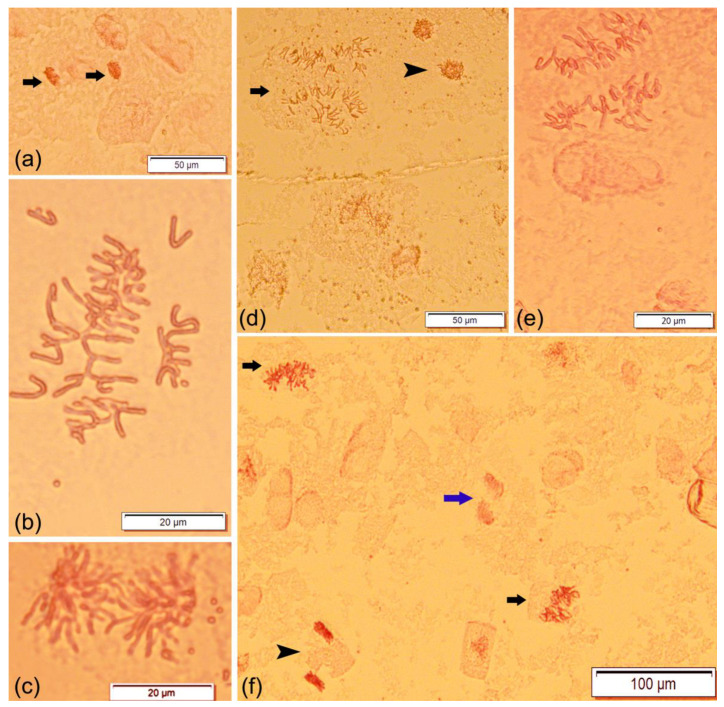
Irregularly dividing cells of triticale cv. ‘Douro’ (2n = 42) in different mitotic phases and stained with aceto-carmine: (**a**) two prophase cells with chromatin stickiness (arrows); (**b**) C-mitosis; (**c**) anaphase with chromatin bridges, stickiness and laggard chromosomes; (**d**) multipolar anaphase (arrow) and a disturbed telophase cell (arrowhead); (**e**) anaphase with disturbed chromosomal orientation and laggard chromosomes; (**f**) two metaphase cells with chromatin stickiness (black arrows), one telophase cell with chromatin stickiness (arrowhead) and anaphase-telophase cell with disturbed orientation (blue arrow).

All treatments showed irregularly dividing cells with one to four types of anomalies per mitotic phase (Figure 3; Table 1).

The types of anomalies presented in Table 1 were the most frequent in all treatments. Additional rare anomalies seen in a few dividing cells, such as ring chromosomes in metaphase, vagrant chromosomes in anaphase (not shown), and telophase cells with disturbed orientation (Figure 3f, blue arrow), were also seen.

The mean number of irregularly dividing cells ascribed to each type of chromosomal or cell cycle anomaly showed statistically significant differences (*p* ˂ 0.05) among treatments (Table 1).

Chromatin stickiness was detected in all treatments and constitutes an irreversible anomaly in the origin of other irregularities, such as chromatin bridges in anaphase, also seen in this work (Table 1). Concerning the individual mitotic phases, the highest mean numbers of dividing cells with chromatin stickiness were observed in the treatments that used Fe or Zn alone (Table 1).

The global results achieved with the root cell cycle analysis suggested that the seed priming treatments performed with the combination of Fe and Zn improved the MI and induced a lower frequency of anomalies, thus ensuring more regular mitosis.

### 2.2. Nucleolar Activity Evaluation

**Figure 4:**
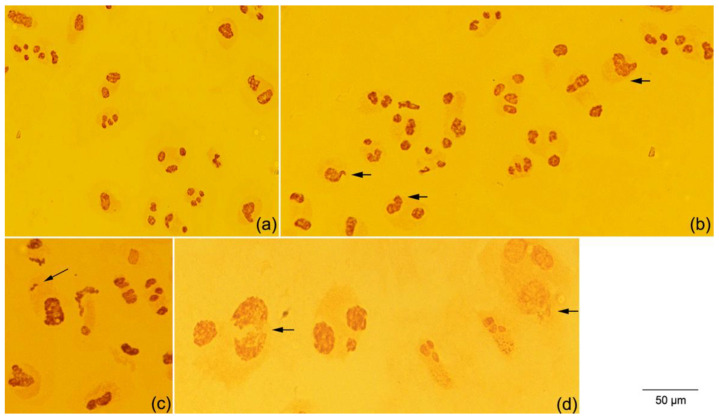
Normal and irregular interphase cells of triticale cv. ‘Douro’ stained with silver nitrate showing: (**a**) one to four nucleoli per nucleus; (**b**) nucleoli with irregular shape (arrows); (**c**) presence of silver-stained particles in the nucleoplasm (arrow); and (**d**) nucleolar disruption (arrows).

The evaluation of the nucleolar activity was performed on a total of 6632 interphase cells and consisted of the following:

(i) Scoring of 5080 interphases with one to four nucleoli per nucleus (Figure 4a);
(ii) Identification of anomalies such as nucleoli with an irregular shape (Figure 4b), silver-stained particles in the nucleoplasm (Figure 4c) or nucleolar disruption (Figure 4d) in 264 interphase cells, corresponding to 3.98% (264/6632 × 100) of the total analysed cells;
(iii) Measure of the nucleolar area in 1288 normal interphase cells presenting one to four nucleoli with a regular shape.

All seed priming treatments showed nuclei with one to four nucleoli (Figure 4a).

**Figure 5:**
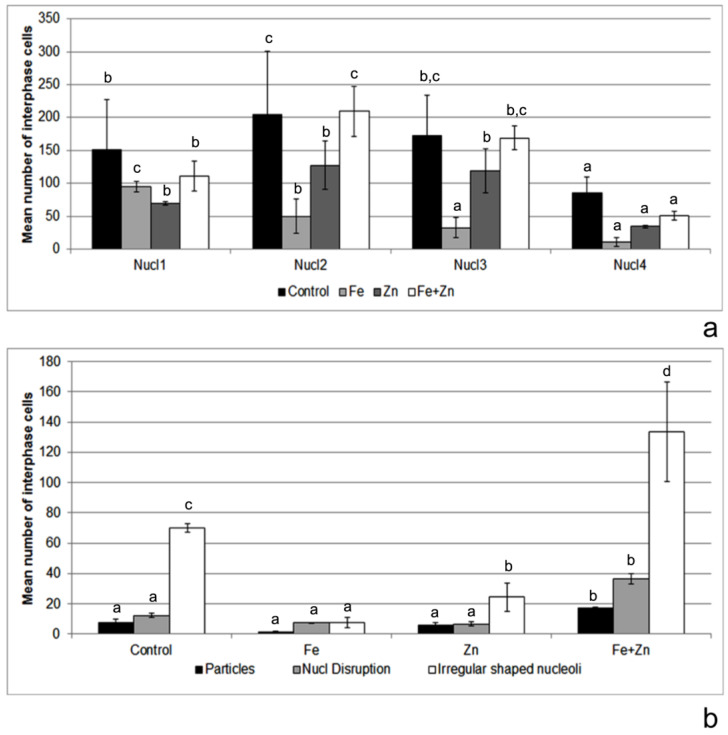
Mean number of interphase cells (±error bar) with (**a**) one to four nucleoli per nucleus; and (**b**) anomalies such as the presence of silver stained particles in the nucleoplasm, nucleolar disruption or nucleoli with an irregular shape, nucleolar disruption or, determined per priming treatment performed in the triticale cv. ‘Douro’ seeds. Different lowercase letters in the columns of the same data series represent statistically significant differences (*p* ˂ 0.05) among treatments.

The mean number of interphase cells with one, two, three or four nucleoli per nucleus showed statistically significant differences (*p* ˂ 0.05) among treatments (Figure 5a). The lowest mean number of interphase cells in all treatments presented four nucleoli per nucleus (Figure 5a). On the other hand, the highest average of interphase cells evidenced two or three nucleoli per nucleus (Figure 5a).

Among the three types of anomalies seen in the interphase cells (Figure 4b–d), nucleoli with an irregular (non-spherical) shape were the most common in all treatments (Figure 5b). The highest mean values of interphase cells with the three types of anomalies were detected in the Fe + Zn treatment. They differed statistically (*p* ˂ 0.05) from the values in the other treatments (Figure 5b).

The nucleolar area was measured in interphase cells with one to four nucleoli with regular shapes from all treatments (Table 2).

The mean nucleolar area decreased significantly (*p* ˂ 0.001) with the increase in the nucleoli number (N) per nucleus (Table 2). In addition, the mean nucleolar area was influenced by the treatment (T) (*p* ˂ 0.001), being higher in roots collected in seeds treated with Fe alone and lower in the ones from the Zn treatment (Table 2).

The nucleolar activity evaluation performed in triticale roots collected from seeds primed with Fe and/or Zn evidenced: (i) no changes in the nucleolar number; (ii) three types of anomalies in 3.98% of the analysed interphase cells (6632), mainly in the Fe + Zn treatment; and (iii) a significant impact of the treatment factor in the nucleolar area which decreased with the increase in the nucleolar number.

## 3. Discussion

Since triticale can grow in acidic soils and tolerate high concentrations of iron and zinc [3,14], we hypothesised that the hexaploid triticale cv. ‘Douro’ would be more tolerant to the 8 mg·L^−1^ dosage of Fe and/or Zn used in seed priming than the bread wheat cv. ‘Jordão’, which showed a certain degree of cytotoxicity previously to these treatments based on the root mitotic cell cycle and nucleolar activity analyses [11,16]. The present results partially confirm this hypothesis.

Triticale seeds have been treated with distilled water (hydropriming), osmotic solutions, melatonin, phytohormones or magneto-priming to improve germination, seedling growth, drought and salt stress tolerance or yield [6,8,9,10]. Agronomic biofortification by seed priming with micronutrient-rich solutions (nutripriming) can be helpful to triticale, given its ability for livestock feed and its potential for developing new food products. Seed nutripriming is cost-effective and can increase crop micronutrient levels [5,22,23]. Additionally, the nutritional quality improved by seed priming can be inherited by unprimed (non-treated) offspring [13]. It can enhance and retain the induced stress tolerance in the ensuing generations [24]. Nevertheless, the micronutrient dosages used in seed priming should be tested for each species or variety to avoid cytotoxicity and phytotoxicity. Cytogenetic approaches such as the root mitotic cell cycle and nucleolar activity evaluation have been proven to be fast and reliable methods to assign the most suitable treatments for seed priming, as demonstrated before in bread wheat [11,16]. These cytogenetic approaches have also helped evaluate the abiotic stress responses in the roots and leaves of other plant species [18,19,21,25,26].

Concerning the mitotic cell cycle analysis, the triticale ‘Douro’ presented a higher mitotic index (MI) in the Fe (47.99%) and Fe + Zn (61.51%) treatments than the bread wheat ‘Jordão’ that showed 33.72% and 29.18% of MI, respectively [16]. This result explains the higher suitability of triticale to grow in acidic soils where the bioavailability of iron and zinc is high. Besides, priming triticale seeds with the 8 mg·L^−1^ Fe + Zn treatment might improve their tolerance to low pH soils.

In the present work, all treatments showed higher mean percentage values of dividing cells with anomalies (DCA) than those found in bread wheat for the respective dosages of Fe and/or Zn [16]. Nevertheless, the lowest average of DCA was registered in the Fe + Zn treatment. Moreover, in most cases, the Fe + Zn treatment presented the lowest mean values of dividing cells with different types of chromosomal or mitotic spindle anomalies. The anomalies observed in the dividing cells were similar to the ones reported for bread wheat primed with Fe and/or Zn [16] and other wheat species under different abiotic stresses [21].

Most of the analysed dividing cells were normal prophases. The highest average of normal prophases was registered in the Fe treatment, suggesting higher cell cycle arresting. The high mean number of normal and/or irregular prophase cells during or after stress is common in plant species under stress [16,21]. The highest mean numbers of irregular metaphase and anaphase cells were also detected in the Fe treatment. The Zn and control treatments showed the highest average of normal telophase cells, suggesting the completion of the cell cycle.

Overall, the cell cycle analysis evidenced that the Fe + Zn and the Zn treatments were less cytotoxic than the Fe treatment to triticale, and the former was not as cytotoxic as it was to bread wheat.

Concerning the nucleolar activity analysis, in all treatments, the maximum number of nucleoli seen per nucleus was four, as expected for hexaploid triticale, given the epigenetic phenomenon of nucleolar dominance where the rRNA genes of rye are silenced by cytosine methylation [27,28,29,30,31]. Only the rRNA genes on the wheat-origin chromosome pair 1B and 6B can organise nucleoli [28]. Therefore, no changes in nucleoli number were induced by the seed priming treatments performed in triticale. As detected in previous studies of nucleolar activity in hexaploid triticale, the average of interphase cells with four nucleoli was lower than the mean values of interphase cells with two and three that were more frequent [28].

The nucleolar size has been positively correlated with cell proliferation, rRNA transcription and transport or protein synthesis [31,32,33,34]. However, the nucleolar number and area changes in response to particular abiotic stress do not always follow the same trend among plant species [11,18,19,21,25,35]. Reducing the nucleolar number and/or area does not imply diminished nucleolar activity or protein synthesis [11,35,36].

The seed priming treatments significantly affected the nucleolar morphology, structure and area. Nucleoli with an irregular shape and disruption were the most common anomalies in the analysed interphase cells. The nucleolar content is released because of nucleolar disruption, which also induces changes in shape. This explains the occurrence of the third type of anomaly detected, the presence of silver-stained particles in the nucleoplasm. Besides, the highest frequencies of interphase cells with two and three nucleoli per nucleus may arise from nucleolar fusion, contributing to an irregular shape. Nucleolar fusion is a common phenomenon in plants where small nucleoli within the nucleus tend to fuse, forming a single larger nucleolus [37]. Similar anomalies were found in bread wheat upon applying the same seed treatments [11]. The three types of anomalies were seen in each seed priming treatment. Only 3.98% of irregular interphase cells were detected, and 2.23% were registered in the Fe + Zn treatment.

Nucleolar stress is a term that has appeared in a few publications [21,36,38,39]. Among its various functions, the nucleolus can regulate the cell cycle and sense intracellular stress, responding with changes in number, morphology, structure, function and size [20,37,38]. In plants, nucleolar stress mechanisms still need to be fully understood [37,38,39]. However, the present work and previous studies we developed in bread, wheat and grapevine allowed us to agree with the usefulness of nucleolar parameters as indicators of plant responses to micronutrient excess or heat stress [11,18,19,26].

The highest mean value of the nucleolar area was found in the Fe treatment. A previous study demonstrated that the plant nucleolus constitutes an unexpected site of massive iron accumulation in healthy cells [40]. These authors explained that the accumulation of a highly reactive and potentially toxic metal in the vicinity of DNA and RNA, despite being risky, could be a defence mechanism to prevent oxidative stress of the nucleic acids rather than a sign of cell dysfunction usually considered in animal cells. The nucleolus may be a sink for excess Fe, where it is stored until required for the various biological processes where it intervenes. However, the excess Fe may induce oxidative stress of the nucleic acids with the subsequent occurrence of DNA strand breaks, which may justify the high frequency of chromosomal and mitotic spindle anomalies seen in the Fe treatment. Also, given the involvement of the nucleolus in cell cycle regulation, the lowest averages of the nucleolar area found in the Zn and Fe + Zn treatments might be correlated with their more regular mitosis relative to the Fe treatment. The Zn treatment also showed the highest average of telophase cells, suggesting mitosis completion. Nonetheless, the Fe + Zn treatment showed the highest MI and the lowest DCA.

Integrating all cytogenetic data allowed us to indicate the combination of Fe and Zn as the most suitable and least cytotoxic seed priming treatment to triticale.

Beyond the in-depth study of the biochemical and molecular mechanisms underlying the nucleolus responses to abiotic stress in plants, and their involvement in cell cycle regularity and regulation, the analysis of the cytogenetic MI and DCA parameters and the nucleolar indicators can provide fast, cost-effective and reliable approaches to defining appropriate dosages of micronutrients to be used in seed nutripriming.

As reported by [1], one of the major challenges to agronomic biofortification, regardless of the strategy used, is the maintenance of micronutrient-dense genotypes through the ensuing generations. In this line of thought, considering the higher tolerance of triticale to high amounts of Fe and/or Zn with an enhanced accumulation of Fe in its nucleolus as seen by the significant enlarging of its area, hexaploid triticale can be used as bridge plant material in wheat breeding programs, acting as a Fe- and Zn-dense progenitor in crosses with wheat. Since hexaploid triticale and wheat have good agronomic performance, as proposed by [1], the development of large populations and further selection can allow the maintenance of Fe- and Zn-dense genotypes through generations.

## 4. Materials and Methods

### 4.1. Plant Material and Seed Priming

The hexaploid triticale cv. ‘Douro’ (2n = 42) is a highly productive and stable cultivar registered in the Portuguese Catalogue of Varieties from 1996 to 2012 after being bred and selected at UTAD for years. In 2020, at the Plant Cytogenomics Lab (UTAD), seeds of the triticale cv. ‘Douro’ were imbibed in aqueous solutions of iron (II) sulphate heptahydrate (FeSO_4_.7H_2_O) and zinc sulphate heptahydrate (ZnSO_4_.7H_2_O) containing 8 mg·L^−1^ of Fe and Zn, respectively, and in distilled water (control, hydroprimed seeds) for 8 h, in the dark, at room temperature. Per treatment, 50 seeds were used. At the end of the treatment, the primed seeds were washed thoroughly with distilled water and allowed to dry to their original moisture content at room temperature.

### 4.2. Collection and Fixation of Roots and Mitotic Spreads Preparation

The seeds of all priming treatments were allowed to germinate in Petri dishes containing filter paper moistened in distilled water, in the dark, at 25 °C.

Roots with a length of 1.5 cm were collected and immediately fixed in a cold (−20 °C) ethanol and acetic acid (3:1, *v*/*v*) solution, freshly prepared. The roots were fixed for at least 16 h and then transferred to a 2% aceto-carmine staining solution for 48 h at 25 °C. The stained roots were used to prepare mitotic spread slides following [41]. The slides were placed at −80 °C overnight. The next day, the glass coverslips were removed, and the slides were air-dried.

### 4.3. Mitotic Cell Cycle and Nucleolar Activity Evaluation

For the mitotic root cell cycle analysis, three slides were used. One drop of immersion oil was loaded on each slide and covered with a 24 × 50 mm glass coverslip. The slides were observed under the optical microscope.

A variable number of interphase and mitotic cells were scored in 50 microscope observation fields per slide. The mitotic phase and chromosomal or cell cycle anomalies were identified in each observation field beyond the cell scoring. Based on these data, we determined per treatment the mitotic index (MI) and the percentage of dividing cells with anomalies (DCA) according to Equations (1) and (2), respectively.

MI (%) = number of dividing cells/number of counted cells × 100,(1)

where the number of counted cells corresponds to the sum of interphase and mitotic cells.

DCA (%) = number of dividing cells with anomalies/number of dividing cells × 100.(2)

We used three slides aged at 60 °C for 2 h for the nucleolar activity evaluation. After cooling, the preparations were incubated in 2× SSC (salt sodium citrate) buffer for 5 min at 55 °C and thoroughly washed in distilled water. In each slide, 100 µL of aqueous solution of 100% silver nitrate (AgNO_3_) was loaded and covered with a nylon coverslip. The staining of nucleoli was allowed at 60 °C for 30 min following [11]. The silver nitrate-stained slides were observed under the optical microscope. Per slide, 50 microscope observation fields were analysed. Per the observation field, a variable number of normal and irregular interphase cells were scored. As normal interphase cells, we considered those presenting one to four integer nucleoli with a spherical shape. We categorised as anomalous interphases those presenting irregularly shaped nucleoli, silver-stained particles in the nucleoplasm or nucleolar disruption.

In triticale, due to the phenomenon of nucleolar dominance, a maximum number of four nucleoli (organised from the chromosome pairs 1B and 6B of wheat origin) per interphase nucleus is expected. The number of interphase cells with a variable nucleoli number (ranging from one to four) was scored per seed priming treatment.

In each seed priming treatment, we also measured the nucleolar area [A (μm^2^) = πr^2^] in normal interphase cells with one to four nucleoli with regular shapes using the Digimizer Image Analysis software (MedCalc, Ostend, Belgium).

Images of cells stained with aceto-carmine and silver nitrate were captured on a microscope Olympus BX41 (Olympus America, Inc., New York, NY, USA) using a CCD digital camera XC-10 (Olympus America, Inc., New York, USA) and the software cellSens (Olympus Soft Imaging Solutions GmbH, Münster, Germany).

### 4.4. Statistical Analyses

Six mitotic spreads per seed priming treatment were performed. Each mitotic spread was done using a single root apex from a different plant. Three mitotic preparations were used for the mitotic cell cycle analysis (*n* = 3), and the other three for nucleolar activity evaluation (*n* = 3). A variable number of cells were scored per preparation. The results are presented as mean ± standard error (S.E.) values per treatment. The statistical analyses of variance (ANOVA) and the post hoc Tukey tests were made with IBM SPSS Statistics v23 (IBM Corporation, New York, NY, USA). The *p*-value significance, due to the different effects and their interaction, was established for probabilities lower than 5% (*p* < 0.05).

## 5. Conclusions

As demonstrated earlier for bread wheat, and in this study, the analysis of the root mitotic cell cycle and nucleolar activity constitute fast, cost-effective and reliable approaches for the definition of cytotoxicity and also suitable dosages of micronutrients to be used in biofortification through seed priming (seed nutripriming).

In this work, the seed priming treatment performed with Fe + Zn increased the mitotic index (MI) and decreased the percentage of dividing cells with anomalies (DCA) relative to the control and the seed priming treatments with Fe or Zn alone. The increase in the MI indicated enhancement of cell division and, hence, root growth, which is particularly needed in dry soils and for seedling growth and establishment. Despite the higher average number of cells in prophase that suggest cell cycle arresting, a reduced DCA was also verified, revealing a higher regularity of mitosis.

The overall cytogenetic data gathered with the cell cycle and nucleolar activity evaluation allowed us to conclude that among the tested seed priming treatments, the combination of Fe + Zn was the most suitable and less cytotoxic to triticale cv. ‘Douro’ could be helpful in its biofortification. Additionally, the Fe + Zn treatment was not as cytotoxic as it was to bread wheat, confirming the higher tolerance of hexaploid triticale to high amounts of these two micronutrients. Nonetheless, as referred before, micronutrient dosages to be used in priming seeds of any crop should be previously tested using the same analyses performed in this work and/or additional and complementary approaches focusing on the agronomic and physiological performances and nutritional content of the biofortified plants and resulting food products. Interestingly, the present findings highlight that triticale accumulates a high amount of Fe and Zn and could be used as bridge material in wheat breeding programmes through interspecific crosses with wheat. Triticale can be used as a Fe- and Zn-dense progenitor in such interspecific crosses to ensure the maintenance of high amounts of these micronutrients in the ensuing generations, constituting one of the major challenges to agronomic biofortification.

## Figures and Tables

**Table 1:** Mean number (±standard error, S.E.) of irregular dividing cells in different mitotic phases showing one to four types of anomalies per priming treatment performed in the triticale cv. ‘Douro’ seeds. Different lowercase letters among each column’s mean values represent statistically significant differences (*p* ˂ 0.05) among treatments.

	Mean Number (±S.E.) of Irregular Dividing Cells in				
	Prophase	Metaphase			Anaphase
Treatment	Chromatin stickiness	Chromatin stickiness	C-mitosis	Disturbed chromosomal orientation	Chromatin bridges
Control	8.33 ± 4.38 a	10.00 ± 9.50 c	5.00 ± 1.53 b	10.00 ± 8.51 b	1.00 ± 1.00 a
Fe	16.00 ± 7.66 b	25.67 ± 6.12 d	3.33 ± 1.67 b	35.33 ± 2.33 c	9.00 ± 1.53 b
Zn	7.00 ± 3.87 a	1.00 ± 0.00 a	4.00 ± 0.58 b	2.33 ± 1.20 a	1.67 ± 0.33 a
Fe + Zn	13.83 ± 7.67 b	5.67 ± 3.28 b	1.67 ± 0.67 a	1.33 ± 1.33 a	0.67 ± 0.67 a
*p*-value	<0.05	<0.05	<0.05	<0.05	<0.05
Mean Number (±S.E.) of Irregular Dividing Cells in					
	Anaphase				Telophase
Treatment	Laggard chromosomes	Disturbed cell polarity	Disturbed chromosomal orientation and stickiness		Chromatin stickiness
Control	1.67 ± 1.20 b	2.00 ± 0.00 b	2.67 ± 2.19 b		1.00 ± 0.37 b
Fe	4.00 ± 0.58 c	9.67 ± 2.85 d	5.33 ± 1.76 c		1.67 ± 0.76 b
Zn	0.33 ± 0.33 a	3.33 ± 1.20 c	1.67 ± 1.20 b		2.33 ± 1.09 b
Fe + Zn	0.67 ± 0.67 a	0.00 ± 0.00 a	0.00 ± 0.00 a		0.33 ± 0.21 a
*p*-value	<0.05	<0.05	<0.05		<0.05

**Table 2:** Mean values (±S.E.) of nucleolar area (μm^2^) determined in normal interphase cells showing one to four nucleoli (N) with regular shape, per priming treatment (T) performed in the triticale cv. ‘Douro’ seeds. Different lowercase letters among the mean values of the nucleolar area represent statistically significant differences (*p* ˂ 0.05) among interphase cells with different nucleolar numbers (N) and treatments (T). The number of interphase cells where the nucleolar area was measured is presented between brackets.

		Nucleolar Area (μm^2^)
Nucleoli number (N)	1	81.95 ± 3.32 d (94 cells)
	2	65.28 ± 2.11 c (379 cells)
	3	50.68 ± 2.10 b (483 cells)
	4	42.11 ± 2.71 a (332 cells)
Treatment (T)	Control	68.53 ± 1.92 b (484 cells)
	8 mg·L^−1^ Fe	107.60 ± 14.49 c (92 cells)
	8 mg·L^−1^ Zn	59.38 ± 3.44 a (150 cells)
	8 mg·L^−1^ Fe + 8 mg·L^−1^ Zn	63.00 ± 2.10 a,b (562 cells)
ANOVA *p*-value	N	<0.001
	T	<0.001
	N × T interaction	<0.001

## Data Availability

Not applicable.

## References

[1] Stangulis J.C.R., Knez M. (2022). Biofortification of major crop plants with iron and zinc—Achievements and future directions. Plant Soil.

[2] Farooq M., Wahid A., Kadambot H., Siddiques M. (2012). Micronutrient application through seed treatments—A review. J. Soil Sci. Plant Nutr..

[3] Soltani E., Soltani A. (2015). Meta-analysis of seed priming effects on seed germination, seedling emergence and crop yield: Iranian studies. Int. J. Plant Prod..

[4] Lutts S., Benincasa P., Wojtyla L., Kubala S.S., Pace R., Lechowska K., Quinet M., Garnczarska M., Araújo S., Balestrazzi A. (2016). Seed priming: New comprehensive approaches for an old empirical technique. New Challenges in Seed Biology-Basic and Translational Research Driving Seed Technology.

[5] Carvalho A., Reis S., Pavia I., Lima-Brito J.E. (2019). Influence of seed priming with iron and/or zinc in the nucleolar activity and protein content of bread wheat. Protoplasma.

[6] Ramzan Y., Hafeez M.B., Khan S., Nadeem M., Saleem-Ur-Rahman, Batool S., Ahmad J. (2020). Biofortification with Zinc and Iron improves the grain quality and yield of wheat crop. Int. J. Plant Prod..

[7] Baltazar M., Oppolzer D., Carvalho A., Gouvinhas I., Ferreira L., Barros A., Lima-Brito J. (2023). Hydropriming and nutripriming of bread wheat seeds improved the flour’s nutritional value of the first unprimed offspring. Plants.

[8] Feng Z., Qi Z., Du D., Zhang M., Zhao A., Hu Z., Xin M., Yao Y., Peng H., Sun Q. (2019). Characterization of a new hexaploid triticale 6D(6A) substitution line with increasing grain weight and decreased spikelet number. Crop J..

[9] Makowska A., Waśkiewicz A., Chudy S. (2020). Lignans in triticale grain and triticale products. J. Cereal Sci..

[10] Blum A. (2014). The abiotic stress response and adaptation of triticale—A review. Cereal Res. Commun..

[11] Zhu F. (2018). Triticale: Nutritional composition and food uses. Food Chem..

[12] Yagmur M., Kaydan D. (2008). Alleviation of osmotic stress of water and salt in germination and seedling growth of triticale with seed priming treatments. Afr. J. Biotechnol..

[13] Alvarez J., Martinez E., Diezma B. (2021). Application of hyperspectral imaging in the assessment of drought and salt stress in magneto-primed triticale seeds. Plants.

[14] Guo Y., Li D., Liu L., Sun H., Zhu L., Zhang K., Zhao H., Zhang Y., Li A., Bai Z. (2022). Seed priming with melatonin promotes seed germination and seedling growth of *Triticale hexaploide* L. under PEG-6000 induced drought stress. Front. Plant Sci..

[15] Camargo C.E.D.O., Felício J.C., De Freitas J.G., Filho A.W.P.F. (1988). Tolerância de trigo, triticale e centeio a diferentes níveis de ferro em solução nutritiva. Bragantia.

[16] Costa H.T.D. (2015). Concentração de Metais Pesados nos Solos Utilizados Para Agricultura Urbana na Cidade de Lisboa. Master’s Thesis.

[17] Reis S., Pavia I., Carvalho A., Moutinho-Pereira J., Correia C., Lima-Brito J. (2018). Seed priming with Iron and Zinc in bread wheat: Effects in germination, mitosis and grain yield. Protoplasma.

[18] Baltazar M., Reis S., Carvalho A., Lima-Brito J. (2021). Cytological and yield-related analyses in offspring of primed bread wheat seeds. Genet. Resour. Crop Evol..

[19] Castro C., Carvalho A., Pavia I., Bacelar E., Lima-Brito J. (2021). Development of grapevine plants under hydroponic Copper-enriched solutions induced morpho-histological, biochemical and cytogenetic changes. Plant Physiol. Biochem..

[20] Castro C., Carvalho A., Pavia I., Bacelar E., Lima-Brito J. (2021). Grapevine varieties with differential tolerance to Zinc analysed by morpho-histological and cytogenetic approaches. Sci. Hortic..

[21] Boulon S., Westman B.J., Hutten S., Boisvert F.-M., Lamond A.I. (2010). The nucleolus under stress. Mol. Cell.

[22] Pekol S., Baloğlu M.C., Çelik Altunoğlu Y. (2016). Evaluation of genotoxic and cytotoxic effects of environmental stress in wheat species with different ploidy levels. Turk. J. Biol..

[23] Cakmak I. (2008). Enrichment of cereal grains with zinc: Agronomic or genetic biofortification?. Plant Soil..

[24] Sheteiwy M.S., Guan Y., Cao D., Li J., Nawaz A., Hu Q., Hu W., Ning M., Hu J. (2015). Seed priming with polyethylene glycol regulating the physiological and molecular mechanism in rice (*Oryza sativa* L.) under nano-ZnO stress. Sci. Rep..

[25] Louis N., Dhankher O.P., Puthur J.T. (2023). Seed priming can enhance and retain stress tolerance in ensuing generations by inducing epigenetic changes and trans-generational memory. Physiol. Plant.

[26] Teerarak M., Bhinija K., Thitavasanta S., Laosinwattana C. (2009). The impact of sodium chloride on root growth, cell division, and interphase silver-stained nucleolar organizer regions (AgNORs) in root tip cells of *Allium cepa* L. Sci. Hortic..

[27] Carvalho A., Leal F., Matos M., Lima-Brito J. (2018). Effects of heat stress in the leaf mitotic cell cycle and chromosomes of four wine-producing grapevine varieties. Protoplasma.

[28] Castilho A., Neves N., Rufini-Castiglione M., Viegas W., Heslop-Harrison J. (1999). 5-Methylcytosine distribution and genome organization in triticale before and after treatment with 5-azacytidine. J. Cell Sci..

[29] Lima-Brito J., Carvalho A., Matos C., Heslop-Harrison P., Guedes-Pinto H. (2005). rRNA gene expression and location in triticale assayed by silver staining and in situ hybridisation techniques. Plant Breed. Seed Sci..

[30] Viegas W.S., Silva M., Neves N., Pereira M.S. (2007). Epigenetics: The Functional Memory of Ribosomal Genes. A Portrait of State-of-the-Art Research at the Technical University of Lisbon.

[31] Georgiev S. (2008). Gene expression and nucleolar dominance in hexaploid triticale and *T. aestivum*. Biotechnol. Biotechnol. Equip..

[32] Handa H., Kanamori H., Tanaka T., Murata K., Kobayashi F., Robinson S.J., Koh C.S., Pozniak C.J., Sharpe A.G., Paux E. (2018). Structural features of two major nucleolar organizer regions (NORs), Nor-B1 and Nor-B2, and chromosome-specific rRNA gene expression in wheat. Plant J..

[33] Kwiatkowska M., Maszewski J. (1985). Functional and structural heterogeneity of nucleoli: The dependence of the activity of transport of newly synthesized rRNA on nucleolar size and phase of the cell cycle. Folia Histochem. Cytobiol..

[34] Mehta R. (1995). The potential for the use of cell proliferation and oncogene expression as intermediate markers during liver carcinogenesis. Cancer Lett..

[35] Tiku V., Jain C., Raz Y., Nakamura S., Heestand B., Liu W., Späth M., Suchiman H.E.D., Müller R.-U., Slagboom P.E. (2017). Small nucleoli are a cellular hallmark of longevity. Nat. Commun..

[36] Bellani L.M., Muccifora S., Giorgetti L. (2014). Response to copper bromide exposure in *Vicia sativa* L. seeds: Analysis of genotoxicity, nucleolar activity and mineral profile. Ecotoxicol. Environ. Saf..

[37] Jordan E.G., Martini G., Bennett M.D., Flavell R.B. (1982). Nucleolar fusion in wheat. J. Cell Sci..

[38] Kalinina N.O., Makarova S., Makhotenko A., Love A.J., Taliansky M. (2018). The multiple functions of the nucleolus in plant development, disease and stress responses. Front. Plant Sci..

[39] Ohbayashi I., Sugiyama M. (2018). Plant nucleolar stress response, a new face in the NAC-dependent cellular stress responses. Front. Plant. Sci..

[40] Roschzttardtz H., Grillet L., Isaure M.-P., Conéjéro G., Ortega R., Curie C., Mari S. (2011). Plant Cell Nucleolus as a Hot Spot for Iron. J. Biol. Chem..

[41] Lima-Brito J., Guedes-Pinto H., Harrison G.E., Heslop-Harrison P. (1996). Chromosome identification and nuclear architecture in triticale x tritordeum F_1_ hybrids. J. Exp. Bot..

